# Thoracoscopic resection of a giant esophageal schwannoma: A case report and review of literature

**DOI:** 10.1097/MD.0000000000039507

**Published:** 2024-08-30

**Authors:** Shu Chen, Yixuan Zhao, Yinghao Zhao

**Affiliations:** a Department of Thoracic Surgery, the Second Hospital of Jilin University, Jilin, China; b Department of Ultrasound Medicine, the Second Hospital of Jilin University, Jilin, China.

**Keywords:** artificial pneumothorax, esophageal schwannoma, VATS

## Abstract

**Introduction:**

Benign esophageal tumors are uncommon, accounting for approximately 2% of esophageal tumors. Esophageal schwannoma is a much rarer solid tumor with few cases reported in the literature. Open surgery is the surgical approach of choice for the treatment of esophageal tumors. With the advent of thoracoscopy, more and more countries are adopting a thoracoscopic approach to treat esophageal tumors, but there is still no clear surgical standard or modality for the treatment of esophageal tumors.

**Patient concerns:**

A 50-year-old woman was admitted to our hospital. Over the past 2 months, her clinical presentation has included progressively worse swallowing disorder and weight loss. Gastroscopy showed an elevated lesion with a smooth surface visible 18 cm out from the incisors. An electron circumferential ultrasound endoscopy showed a hemispherical bulge with a smooth surface 18 to 23 cm from the incisor; the bulge originated from the intrinsic muscular layer and showed a heterogeneous mixed moderate ultrasound with a little blood flow signal and blue-green elastography in 1 of the sections measuring approximately 4 cm × 3 cm. Chest computed tomography (CT) showed a mass-like soft tissue shadow in the upper esophagus measuring approximately 39 mm × 34 mm, with a CT The lumen was compressed and narrowed, and the lumen of the upper part of the lesion was dilated, and the adjacent trachea was compressed and displaced to the right.

**Interventions:**

After completion of the examination, assisted by artificial pneumothorax and thoracoscopic resection of esophageal masses were performed.

**Diagnosis and Outcomes:**

Postoperative pathology report: Mesenchymal-derived tumor (esophagus), combined with immunohistochemical staining results and morphologic features supported schwannoma. The patient’s postoperative course was calm. The patient’s postoperative dysphagia subsided.

**Conclusion:**

We describe a case of successful treatment of a schwannoma of the upper esophagus using artificial pneumothorax-assisted VATS. The combined use of Sox10 and S100 helps to improve the sensitivity and specificity of schwannoma diagnosis. Damage to the esophageal lining was avoided by mixed thoracoscopic and endoscopic exploration. This approach can also be applied to benign esophageal tumors in the thoracic and subthoracic segments, leading to better minimally invasive results.

## 1. Introduction

The majority of esophageal tumor lesions are esophageal cancer. The benign primary esophageal tumors which account for about 2% of all esophageal tumors are uncommon. Over 80% of benign esophageal tumors present as smooth muscle tumors and less commonly as esophageal schwannoma.^[1,2]^ Esophageal schwannoma are characteristically neurogenic tumors of mediastinal origin. It occurs rarely and is difficult to diagnose by imaging. They are usually benign and exhibit 1 or 2 histological patterns: Antoni A and B.^[3]^ Esophageal schwannoma are usually seen in Asian patients, with a predominance of women and middle-aged (approximately 50–60 years old) patients.^[4]^ One of the most common symptoms is dysphagia. Histological and immunohistochemical studies are required for diagnosis, and surgical resection is the primary treatment for the disease.

**Figure 1.**
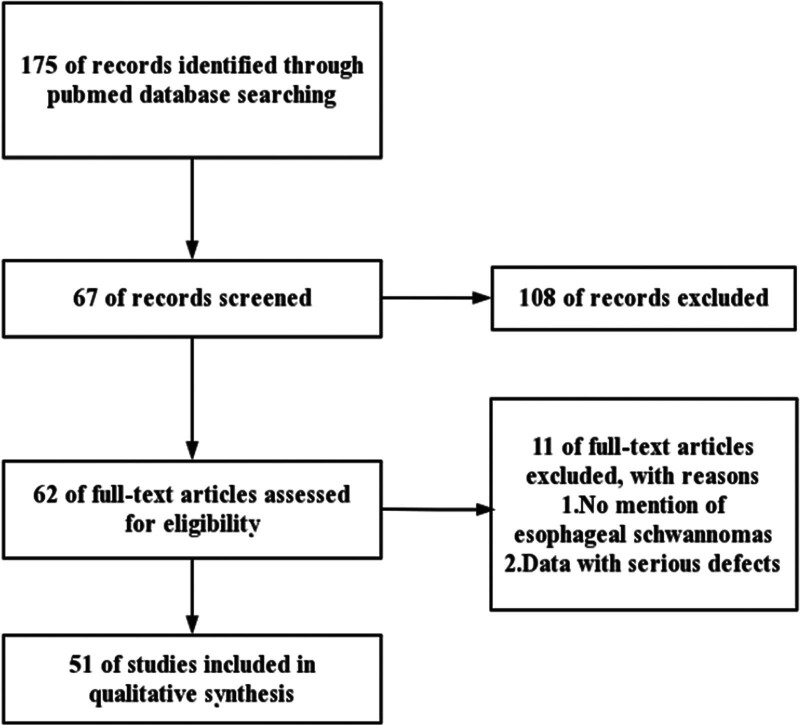
Procedure of retrieval.

A literature search was performed in the PubMed database with the subject terms “(esophageal schwannoma or Esophageal nerve sheath tumor)” and “surgery” and there were 175 studies published on this topic as of December 2022. The reference lists of all full-text retrieved were screened to further identify potentially relevant studies. The detailed search strategy is shown in the study flowchart (Fig. 1). Only 1 investigator reviewed the cases reported in the literature. The following variables were extracted for each case report: author, age, gender, symptoms, surgical procedure, immunohistochemical results, benign and malignant.

Initially, 62 case reports and series were identified in the literature, 11 of which were excluded due to incomplete data according to the previously described methodology, and 51 were finally included. Detailed patient data are available in Table 1.

**Table 1:** Surgical resection of esophageal schwannomas: a review of the literature.

Case	Author	Year	Age	Sex	Symptoms	Surgical approach	Immunohistochemistry	Benign or malignant
1	Kenya Kobayashi	2019	69	M	Dyspnea	Neck approach	S100(+), SOX10(+)	Benign
2	A. Sáncheza	2004	54	M	Dysphagia	Thoracotomy	S100(+), vimentin(+)	Malignant
3	Naoki Miyamoto	2021	70	M	Dysphagia	Thoracotomy	S100(+)	Benign
4	Masahiro Kitada	2013	55	F	Palpitations + swallowing discomfort	Thoracotomy	S100(+)	Benign
5	Hyun Woo Jeon	2014	63/32	M/F	Asymptomatic/intermittent chest pain	Thoracotomy/neck approach	S100(+)	Benign
6	M.V. Matteo	2020	22	M	Dysphagia + acute chest pain	Thoracotomy	S100(+)	Benign
7	Mi Jin Gu	2014	39	M	Dysphagia	VATS	S100(+)	Benign
8	Yajie Zhang	2018	48	F	Dysphagia	RATS	S100(+)	Benign
9	Yoshinori Iwata	2018	74	F	Loss of consciousness	Thoracotomy, neck approach	S100(+)	Benign
10	Kazuki Moro	2017	66	M	Dysphagia	Neck approach	S100(+)	Benign
11	Kento Tomizawa	2020	30	F	Dysphagia	Thoracotomy	S100(+), MIB-1 LI(25%)	Malignant
12	Dongbin Ahn	2014	36	F	Neck mass	Neck approach	N	Benign
13	Byonggu An	2018	74	F	Asymptomatic	VATS	S100(+)	Benign
14	Ayako Tomono	2015	59	F	Dysphagia	Thoracotomy	S100(+)	Benign
15	A. Basoglu	2006	54	F	Dysphagia + neck mass	Cervical and abdominal approach	S100(+), vimentin(+)	Malignant
16	H.-C. Chen	2006	73	F	Dyspnea + dysphagia	VATS	S100(+)	Benign
17	Roman Dutta	2009	52	F	Dysphagia	Thoracotomy	S100(+)	Benign
18	Timothy J. Eberlein	1992	62	M	dysphagia	Neck approach	S100(+)	Benign
19	Zi-ye Gao	2020	47	F	dysphagia	ESD	S100(+)	Benign
20	Shelly S. Choo	2011	22	M	Dyspnea + dysphagia	Thoracotomy	S100(+)	Benign
21	Harbi Khalayleh	2021	61	F	Dysphagia	VATS	S100(+)	Benign
22	Hiroshi Iwata	1993	56	F	Asymptomatic	Thoracotomy	S100(+)	Benign
23	Xiankai Chen	2016	46/42/58	M/F/F	Dysphagia	VATS/thoracotomy/thoracotomy	S100(+)	Benign
24	Edmund S. Kassis	2012	65	M	Asymptomatic	Thoracotomy	S100(+)	Benign
25	Katarzyna Kozak	2015	37	F	Dysphagia	Thoracotomy	S100(+), GFAP(+)	Benign
26	Biswajit Mishra	2016	27	F	Dysphagia	Thoracotomy	S100(+), Ki67(2–3%)	Malignant
27	Nobuyuki Kobayashi	2000	62	F	Asymptomatic	Thoracotomy	S100(+)	Benign
28	Tieqin Liu	2012	62	F	Dysphagia + dyspnea	Thoracotomy	S100(+)	Benign
29	Jad A. Degheili	2019	50	F	Dyspnea	Thoracotomy	S100(+)	Benign
30	Emilio Sanchez-wGarcia Ramos	2019	40	F	Dysphagia	Neck approach	S100(+)	Benign
31	Usman Khan	2021	60	F	Dysphagia	VATS	N	N
32	T. Makino	2011	72	M	Asymptomatic	VATS	S100(+)	Benign
33	T Manger	2000	60	F	Dysphagia	Thoracotomy	S100(+), vimentin(+)	Malignant
34	A. Matsuki	2009	73	F	Progressive speech impairment and chest pain	Thoracotomy	S100(+)	Benign
35	Chun-Xiao Wu	2020	67	F	Dysphagia + dyspnea	Thoracotomy	S100(+)	Benign
36	Shinjiro Mizuguchi	2008	29	F	Dyspnea	VATS	S100(+)	Benign
37	Katsutoshi Murase	2000	49	F	Asymptomatic	Thoracotomy	S100(+), Neuron specific enolase(+)	Malignant
38	L. Zhu	2018	55	F	Dysphagia + pain behind the sternum	Thoracotomy	S100(+)	Benign
39	Masakazu Ohno	2000	49	F	Dysphagia	Thoracotomy	S100(+)	Benign
40	Donglei Liu	2015	62	F	Aphasis	Thoracotomy	S100(+)	Benign
41	Bernard J. Park	2005	33	F	Reduced breathing sounds in the right chest and mild weakness in the right upper limb	Thoracotomy	S100(+)	Benign
42	Reijiro Saito	2000	63	F	Dysphagia	Thoracotomy	S100(+)	Benign
43	Yuto Shimamura	2016	57	M	Intermittent acid reflux	ESD	S100(+)	Benign
44	Toshiteru Tokunaga	2007	46	F	Dyspnea + dysphagia	Thoracotomy	S100(+)	Benign
45	Arvind J. Trindade	2017	54	M	Asymptomatic	ESD	S100(+)	Benign
46	Shaohua Wang	2011	44	F	Dysphagia	Thoracotomy	S100(+), vimentin(+)	Malignant
47	Takayoshi Watanabe	2016	39	F	Dysphagia	VATS	S100(+)	Benign
48	Bin Li	2020	59/51/49	M/F/M	Upper abdominal distension/Discomfort in the upper abdomen/dysphagia	ESD	S100(+)	Benign
49	Tian-Yi Wang	2021	62	M	Dysphagia	VATS	S100(+)	Benign
50	Yu Onodera	2017	47	F	Dysphagia	VATS	S100(+)	Benign
51	Ho Young Yoon	2008	65	M	Neck mass + dysphagia	N	S100(+)	Benign

M = male, F = female, VATS = video-assisted thoracoscopic surgery, RATS = robot-assisted thoracoscopic surgery, ESD = endoscopic submucosal dissection, N = not mentioned.

This study focuses on the analysis of a 50-year-old female patient who presented with progressive dysphagia and weight loss, with a preliminary preoperative diagnosis of esophageal smooth muscle tumor and a preliminary postoperative diagnosis of esophageal schwannoma. The aim of this study was to investigate the applicability of manual pneumothorax-assisted thoracoscopic resection of giant esophageal schwannoma and the pathological features of esophageal schwannoma, and to review the relevant literature.

## 2. Methods

The patient was positioned in the left lateral position on the operating table with the operator and scope hand on the left side of the patient and the assistant on the right side of the patient. Under general anesthesia, both lungs are ventilated using a single-lumen endotracheal tube. The skin of the 8th intercostal space was incised 1 cm from the axillary midline, and a poke card was placed and normal oxygen saturation was maintained after a short period of low tidal volume (250 mL/min) single-lumen tracheal intubation. A right-sided artificial pneumothorax was created by injecting 8 to 12 cm H_2_O positive pressure carbon dioxide (CO_2_) through the poke card and a thoracoscope was placed. Incisions of 1 cm, 0.5 cm and 1 cm were made in the anterior axillary line of the 4th intercostal space, the posterior mid-axillary line of the 6th intercostal space and the posterior axillary line of the 7th intercostal space, respectively, and poke cards were placed. Normal tidal volume (400–500 mL/min) was restored. The location of the esophageal tumor was explored thoracoscopically, and the mediastinal pleura was opened by ultrasonic knife to bluntly separate the esophageal tumor without damaging the esophageal lining. All specimens were placed in a specimen bag, and the 4th rib incision was extended until the specimen was removed. The drainage tube was disposed of through the right 7/8th rib incision and the incision was sutured as well as the drainage tube was fixed. The procedure was concluded after endoscopic exploration of the esophageal lining without damage.

Prior to surgery, the patient and family were informed of the benefits and risks of this new approach. In case of intraoperative rupture of large blood vessels or endothelial injury, alternative surgical approaches (open access or triple-incision radical esophageal cancer surgery) may be performed. Written informed consent was obtained from patients and their families, and ethical approval was obtained from the Research Ethics Committee of the Second Hospital of Jilin University.

## 3. Case report

**Figure 2.**
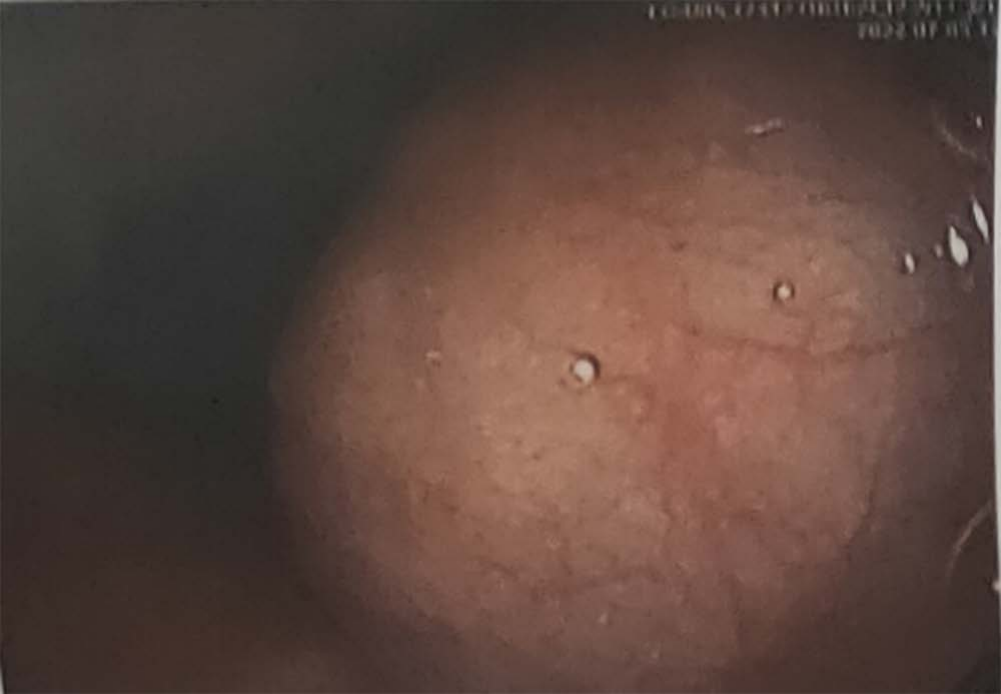
An electron circumferential ultrasound endoscopy showed a hemispherical bulge with a smooth surface 18 to 23 cm from the incisor; the bulge originated from the intrinsic muscular layer and showed a heterogeneous mixed moderate ultrasound with a little blood flow signal and blue-green elastography in 1 of the sections measuring approximately 4 cm × 3 cm.

**Figure 3.**
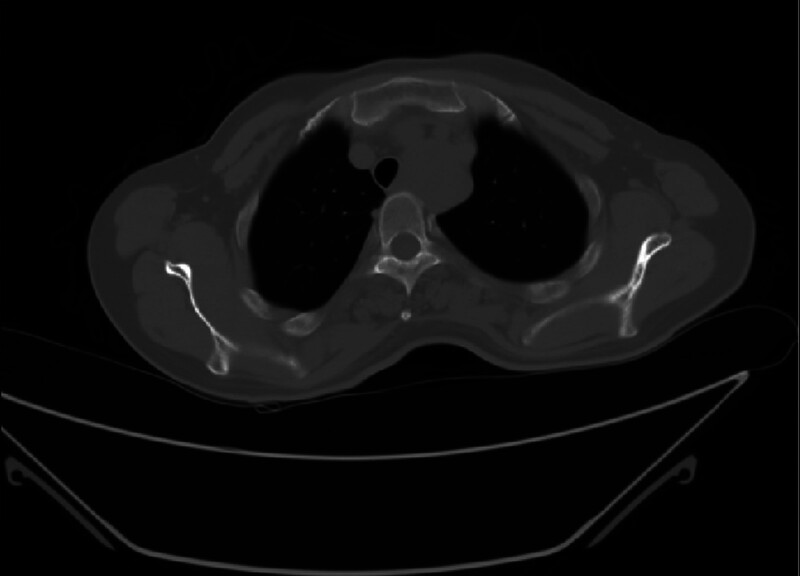
Chest computed tomography (CT) showed a mass-like soft tissue shadow in the upper esophagus measuring approximately 39 mm × 34 mm.

**Figure 4.**
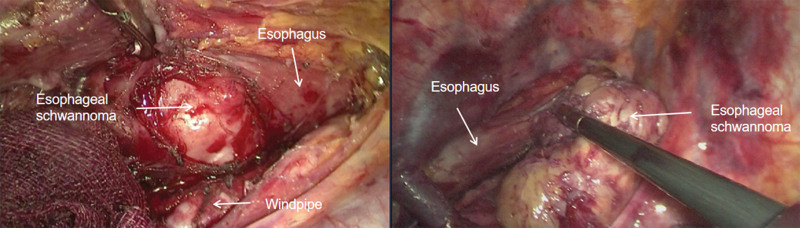
Images during surgery.

**Figure 5.**
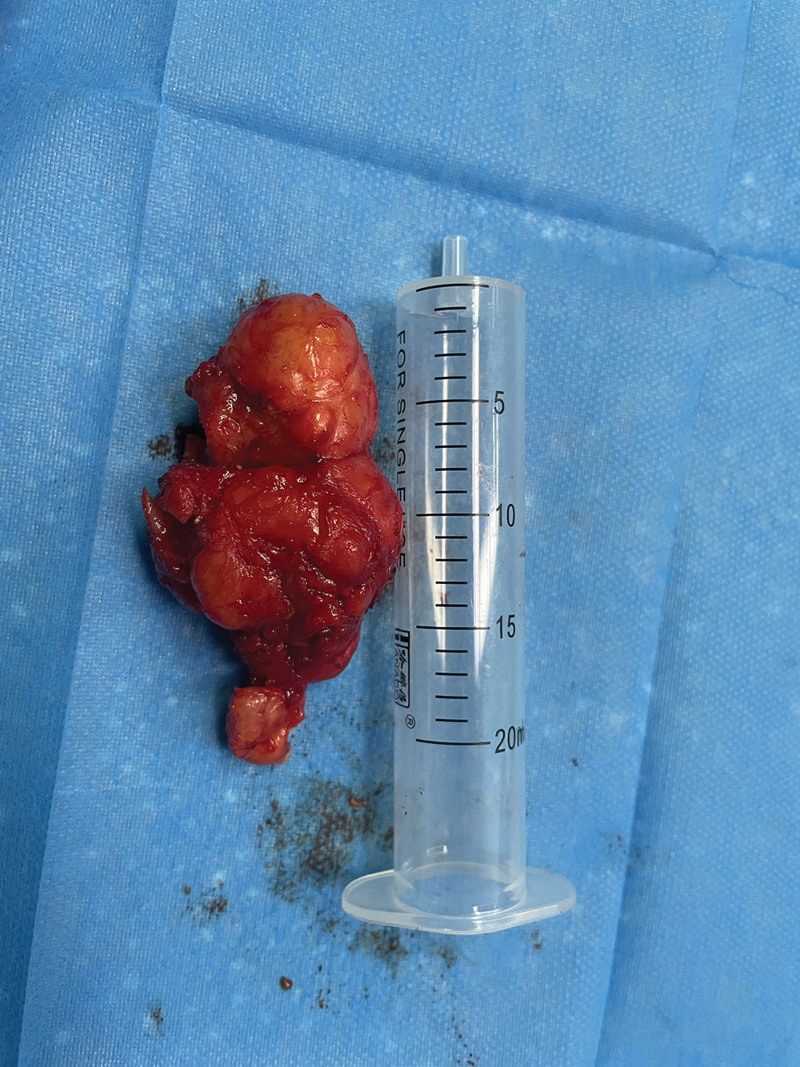
A solid mass of 7.0 cm × 4.0 cm × 4.5 cm.

**Figure 6.**
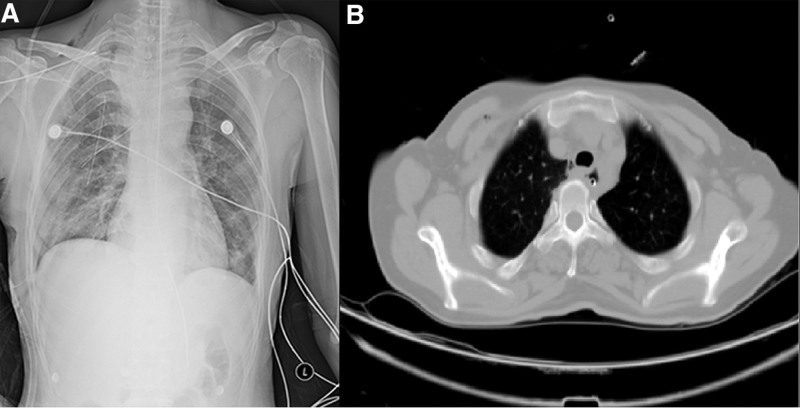
(A) On the first postoperative day, bedside orthographic film; (B) chest CT on day 5 after surgery.

A 50-year-old woman was admitted to our hospital. Over the past 2 months, her clinical presentation has included progressively worse swallowing disorder and weight loss. Physical examination did not reveal any problems. Gastroscopy showed an elevated lesion with a smooth surface visible 18 cm out from the incisors. An electron circumferential ultrasound endoscopy showed a hemispherical bulge with a smooth surface 18 to 23 cm from the incisor; the bulge originated from the intrinsic muscular layer and showed a heterogeneous mixed moderate ultrasound with a little blood flow signal and blue-green elastography in 1 of the sections measuring approximately 4 cm × 3 cm (Fig. 2). Chest computed tomography (CT) showed a mass-like soft tissue shadow in the upper esophagus measuring approximately 39 mm × 34 mm (Fig. 3), with a CT The lumen was compressed and narrowed, and the lumen of the upper part of the lesion was dilated, and the adjacent trachea was compressed and displaced to the right. No biopsy was performed due to the anticipated tumor resection. After completion of the examination, thoracoscopic resection of the esophageal mass was performed under general anesthesia with a single-lumen tube tracheal intubation (Fig. 4). A solid mass of 7.0 cm × 4.0 cm × 4.5 cm was removed and sent for pathological examination (Fig. 5). Postoperative pathology report: Mesenchymal-derived tumor (esophagus), combined with immunohistochemical staining results and morphologic features supported schwannoma. Immunohistochemical staining results: CD117(−), NSE(−), Desmin (focal +), CD34(−), S100(+), SMA(−), Ki67 (3% positivity), H-Caldesmon(−), DOG-1(−), SDHB(+), P53(−), SOX10(+), H3K27me3 (+). Bedside chest radiograph on postoperative day 1 showed good bilateral lung expansion and sharp bilateral rib diaphragm angles (Fig. 6A). The gastric tube was removed after a repeat chest CT on postoperative day 5 (Fig. 6B). The patient’s postoperative course was calm. The patient’s postoperative dysphagia subsided.

## 4. Discussion

Comparable to other submucosal tumors of the esophagus, including smooth muscle tumors and gastrointestinal mesenchymal tumors (GIST), esophageal schwannoma are usually asymptomatic. The most common symptoms, if symptoms are present, are dysphagia and chest discomfort.^[5]^ Other reported signs and symptoms include chest pain, wheezing, vomiting of blood, cough and palpable neck mass.^[6]^

Esophageal schwannoma are most often seen in the upper, middle, and mediastinal esophagus. It is difficult to diagnose this condition preoperatively, and the final diagnosis is usually determined after resection.^[7]^ There are several morphologic variants of nerve sheath tumors, namely conventional, cellular, microcystic/reticular, plexiform, and melanotic nerve sheath tumors.^[8]^ Esophageal schwannoma usually occur more often in women than in men, in a ratio of 4 to 1, especially between the ages of 50 and 60.^[5]^ There have also been reports of patients with malignant schwannoma, but such cases are extremely rare.^[9]^ Some of the symptoms of this disease include dysphagia, dyspnea and chest pain, and may develop and worsen as the size of the esophageal schwannoma increases.^[10]^

It is necessary in our current situation to obtain biopsy results by esophagogastric endoscopy to establish the diagnosis. Schwannoma is a kind of submucosal tumor and ultrasound endoscopy guided fine-needle aspiration biopsy has been reported to be useful for both diagnosis and treatment.^[11]^ Certain investigators have suggested that the current biopsy technique using endoscopic ultrasound-guided fine-needle aspiration has a diagnostic accuracy of 52% to 86% for submucosal esophageal tumors.^[12,13]^ Esophageal schwannoma cells were positive for S100 protein but negative for smooth muscle markers such as actin and desmin, which were positive in smooth muscle tumors, while CD34 and CD117 were characteristically positive in GIST.^[14]^ Recent studies have shown that Sox10 is a potential molecular biological marker for the diagnosis and differentiation of some tumors of the nervous system. Sox10 is consistently expressed in gastrointestinal nerve sheath tumors and can distinguish them from mesenchymal tumors that are interstitially S100 protein positive. Sox10 is superior to S100 as a molecular marker in terms of sensitivity and specificity for the differential diagnosis of schwannoma and fibrous meningiomas.^[15,16]^ Studies have also shown that Sox10 is more specific than S100 for tumors of neural crest origin: Sox10 (99% specificity) and S100 (91% specificity).^[17]^ The combined use of Sox10 and S100 helps to improve the sensitivity and specificity of schwannoma diagnosis.

Benign esophageal schwannoma typically require only tumor removal, not complete resection. Identifying the submucosal surgical plane is challenging due to its size; however, ensuring the integrity of the mucosa is paramount.^[18]^ There is a clear preference in the literature for schwannoma in the upper esophagus, so a right thoracic approach is usually chosen.^[1,2,6,19]^ Currently, VATS is becoming increasingly popular because it is less painful and has a shorter recovery time than open thoracic surgery. Takayoshi Watanabe et al reported the difficulty of resecting esophageal nerve sheath tumors larger than 5 cm using a VATS approach and the need to convert from resection to subtotal esophagectomy.^[20]^ Minimally invasive resection of benign esophageal tumors is technically safe and has a shorter hospital stay compared to open surgery, a retrospective study suggests. Although the exact size threshold could not be determined, most tumors larger than 7 cm were removed by open thoracotomy.^[21]^ Nonetheless, there are limitations to the possibility of thoracoscopic resection of esophageal schwannoma due to the size and location of the tumor. It has been a challenge to completely resect the huge tumor without compromising the mucosal integrity due to the limitation of 2-dimensional view and range of motion of conventional thoracoscopic tools.

A thoracoscopic esophagectomy in the prone position has been reported as a safe method of treating esophageal cancer in the thoracic segment.^[22,23]^ The thoracic/subthoracic esophagus is automatically detached from the descending aorta by gravity without ligature after mobilization of the surrounding tissues.^[24]^ The narrow gap between the upper thoracic esophagus, trachea and artery cannot be achieved through a prone position. In contrast, the artificial pneumothorax assists in increasing the tissue gap through CO_2_ filling, which facilitates intraoperative reduction of surrounding tissue damage.

Most recently, there has been improved visibility and flexibility in esophageal surgery provided by RATS using the da Vinci Surgical System. Hecheng Li et al have performed resection of a giant esophageal nerve sheath tumor located in the posterior mediastinum by the da Vinci Surgical System. The robotic approach offers advantages over conventional thoracoscopic systems, including wrist-like motion of the instruments, 3-dimensional vision, and ergonomic comfort for the surgeon. These features facilitate a combination of sharp and blunt dissection in a narrow space, subsequently offering the possibility of complete tumor removal without interrupting the peritoneum and the surrounding esophageal mucosa.^[25]^ Nevertheless, robotic surgery is relatively costly and unaffordable for the general public.

In this case, the esophageal tumor was close to the incisors and was considered to be a tumor of the upper esophagus. Although the location was on the left side of the thoracic cavity, a thoracoscopic right-sided approach was still used, and a cervicothoracic-abdominal triple incision was performed for radical esophageal cancer if necessary. Resection of submucosal esophageal tumors through a thoracoscopic approach can sometimes lead to accidental opening of the esophageal mucosa.^[22]^ To reduce the possible risk of intraoperative esophageal mucosal injury, we chose to ensure the integrity of the esophageal lining through thoracoscopic surgery and simultaneous intraoperative upper gastrointestinal endoscopy. This hybrid endoscopic and thoracoscopic approach allows the surgeon to easily perform tumor debulking without accidentally opening the esophageal mucosa.

## 5. Conclusion

In conclusion, we describe a case of successful treatment of a schwannoma of the upper esophagus using artificial pneumothorax-assisted VATS. The combined use of Sox10 and S100 helps to improve the sensitivity and specificity of schwannoma diagnosis. Damage to the esophageal lining was avoided by mixed thoracoscopic and endoscopic exploration. This approach can also be applied to benign esophageal tumors in the thoracic and subthoracic segments, leading to better minimally invasive results.

## Acknowledgments

We would like to thank the researchers and study participants for their contributions.

## Author contributions

**Conceptualization:** Shu Chen, Yinghao Zhao.

**Supervision:** Yinghao Zhao.

**Validation:** Yinghao Zhao.

**Visualization:** Yinghao Zhao.

**Writing – original draft:** Yixuan Zhao.

**Writing – review & editing:** Shu Chen.
